# Assessment of subclinical left ventricle myocardial dysfunction using global myocardial work in type 2 diabetes mellitus patients with preserved left ventricle ejection fraction

**DOI:** 10.1186/s13098-021-00781-x

**Published:** 2022-01-28

**Authors:** Tao Wang, Li Li, Jun Huang, Li Fan

**Affiliations:** 1 Department of Radiology, The Affiliated Changzhou No. 2 People’s Hospital with Nanjing Medical University, Changzhou, 213003 China; 2 Department of Pediatrics, Changzhou Fourth People’s Hospital, Changzhou Tumor Hospital Affiliated to Soochow University, Changzhou, 213003 China; 3 Department of Echocardiography, The Affiliated Changzhou No. 2 People’s Hospital with Nanjing Medical University, Changzhou, 213003 China

**Keywords:** Myocardial work, Global longitudinal strain, Subclinical LV dysfunction, Type 2 diabetes mellitus

## Abstract

**Background:**

The purpose of this study was to assess subclinical left ventricle (LV) myocardial dysfunction using global myocardial work (MW) in type 2 diabetes mellitus (T2DM) patients with preserved left ventricle ejection fraction (LVEF).

**Methods:**

Sixty T2DM patients and 60 normal controls were enrolled in the study. Apical 4-, 3- and 2-chamber views were acquired by two-dimensional echocardiography. Peak systolic myocardial global longitudinal strain (GLS), global myocardial work index (GWI), global constructive work (GCW), global wasted work (GWW), and myocardial work efficiency (GWE) were determined by speckle-tracking echocardiography (STE).

**Results:**

The GLS values in the T2DM patients were significantly lower than those in normal controls (p < 0.001). The GWW in T2DM patients was significantly greater than that in normal controls, while GWI, GCW and GWE was significantly lower (p < 0.001). Receiver operating characteristic (ROC) analysis showed there were no significant different difference between GWW, GWE and GLS in the area under the curves (AUCs). In T2DM patients, fasting plasma glucose was positively correlated with GWW but negatively correlated with GWE, and GLS was negatively correlated with GWI and GCW.

**Conclusion:**

From the research, we found that global MW as new technique could detect the subclinical LV myocardial dysfunction and confirm that the impaired LV function in T2DM patients with preserved LV systolic function.

## Background

The incidence of type 2 diabetes mellitus (T2DM) has increased worldwide in recent years. The development of coronary artery disease induced by T2DM has also increased. In the past few years, cardiovascular disease has become the major cause of morbidity and mortality for T2DM patients [1]. The pathological changes of diabetic-induced cardiomyopathy mainly include cardiomyocyte apoptosis, myocardial fibrosis and necrosis, ultimately leading to systolic and diastolic cardiac dysfunction and heart failure [2, 3]. Therefore, to protect cardiac function, early detection and intervention of the function of the left ventricle (LV) is essential to the prevention and management of diabetic cardiomyopathy.

Many techniques can be used to assess cardiac function, such as cardiovascular magnetic resonance T1 mapping [4], Tc-99 m MIBI gated single-photon emission-computed tomography myocardial perfusion imaging [5], tissue Doppler strain echocardiography [6], and two-dimensional speckle tracking echocardiography (2D-STE) [7, 8]. However, these techniques all have limitations. Like angle dependence in tissue Doppler strain echocardiography, long-term examination in cardiac magnetic resonance imaging and the radioactivity of cardiac radionuclide imaging, 2D-STE has a lower signal-to-noise ratio, weak acoustic windows, and through-plane motion artefacts [9, 10]. However, 2D-STE has become the leading reliable diagnostic technique for the assessment of cardiac function because of its noninvasiveness, convenience, and repeatability. In addition, our group previously reported that LV longitudinal myocardial function detected by longitudinal strain and rotation in T2DM patients with preserved LV ejection fraction (LVEF) was impaired when compared with that in healthy subjects [11]. However, global myocardial work (MW) is a new parameter for 2D-STE that takes into account deformation as well as afterload through interpretation of strain in relation to dynamic noninvasive LV pressure, potentially offering incremental value to myocardial function assessment.

The aim of the research was to explore the incremental value of global MW in the detection of subclinical LV myocardial dysfunction in T2DM patients with preserved LV systolic function.

## Subjects and methods

### Ethical statement

The study conducted according to the Principles of the Declaration of Helsinki and was approved by the human research and ethics committee of the affiliated Changzhou No. 2 people’s hospital with Nanjing Medical University. All of the patients had completed the informed consent forms.

### Study population

Sixty T2DM patients (not well-treated, and it means poor blood glucose control in these T2DM patients before hospitalization) and 60 normal controls of similar age and sex were enrolled for this study and recruited from the hospital. The inclusion criteria for T2DM patients were clinically confirmed in accordance with the World Health Organization criteria [12] and without any history of heart disease (congenital heart disease, coronary artery disease, arterial hypertension, myocardial infarction, cardiomyopathy, valvular disease, atrial fibrillation, thyroid disease, neoplastic disease, or kidney failure), and also without obesity and dyslipidemia. All enrolled subjects were performed with coronary CT or coronary angiography to confirm that they have no coronary artery disease. All the T2DM patients had a LVEF > 55%. Normal controls were recruited from the physical examination in the hospital. In the normal controls, all of the physical and laboratory examination tests for cardiac function, electrocardiogram (ECG), and echocardiography were normal.

### Anthropometric and biochemistry

The sex, age, height, body weight, heart rate (HR) and blood pressure (systolic blood pressure: SBP, diastolic blood pressure: DBP) of all subjects were recorded at the time of the echocardiography examination. Fasting plasma glucose, glycated haemoglobin (HbA1c), total cholesterol (TCH), triglyceride (TG), high density lipoprotein (HDL) and low density lipoprotein (LDL) were measured when the patients were in hospital.

### Conventional 2D Doppler echocardiography

Patients underwent conventional 2D transthoracic Doppler echocardiography with a Vivid E9 equipped with an M5S 3.5–5 MHz transducer (GE Vingmed Ultrasound, Horten, Norway) by an experienced cardiologist. All of the patients were connected to ECG leads. Apical 4-chamber, 2-chamber, and long-axis views of three consecutive cycles with a standard high frame rate (> 45 s^−1^) were stored for offline analysis. The examination of echocardiography and the measurement of fasting plasma glucose, HbA1c, TCH, TG, HDL and LDL were performed in the same day.

Echocardiographic parameters of left atrial diameter (LAd), interventricular septum thickness (IVSd), LV posterior wall thickness (LVPWd) and LV diameter (LVDd) in the end-diastole period, the peak early (E) and late (A) diastolic mitral annular velocities, LV end-diastole volume (LVEDV), LV end-systole volume (LVESV) and LVEF (modified biplane Simpson's method), and the peak early (e′) and late (a′) diastolic annular velocities (obtained by averaging the values at the septum and lateral wall by tissue Doppler imaging: TDI) were measured during the examination.

### Two-dimensional STE

Global longitudinal strain and MW were measured by EchoPAC software (EchoPAC Version: 203, GE Vingmed Ultrasound, Norway).

**Fig. 1:**
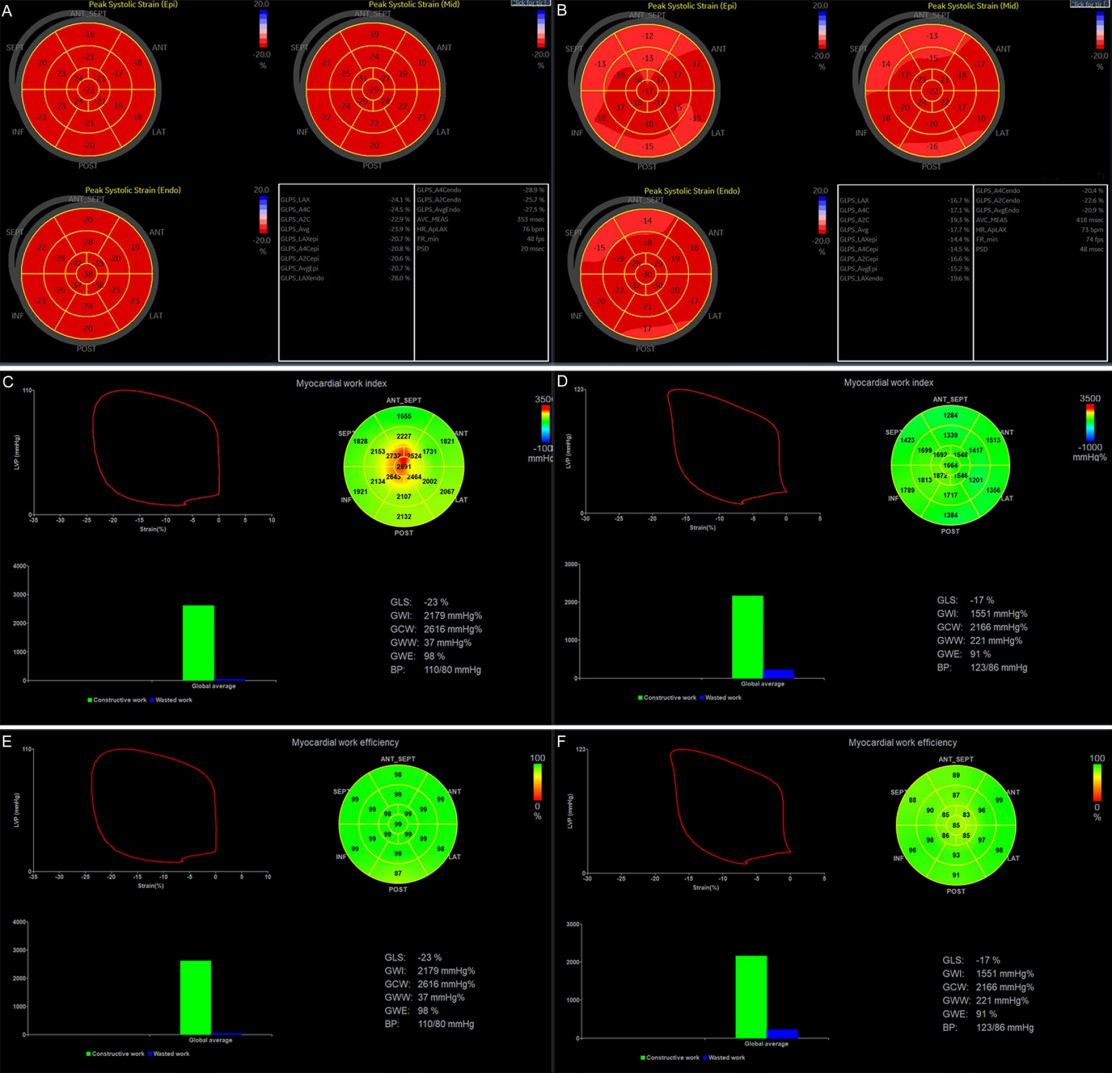
The bull’s eyes of the peak systolic myocardial longitudinal strain of normal controls and T2DM patients (**A** and **B**). global myocardial work of normal controls and T2DM patients (**C**–**F**)

First, the aortic valve closure time was identified from the event timing of the aortic valve spectrum. Then, APLAX, A4C and A2C were used to analyse the apical long-axis, four-chamber and two-chamber views. The LV myocardium was divided into 18 segments, and the global longitudinal strain (GLS) was automatically measured by the software (Fig. 1A and B).

MW and related indices were calculated using a combination of echocardiographic strain data and a noninvasively estimated LV pressure curve. Blood pressure was input into the software, and “Advanced” was clicked. The software constructed a non-invasive LV pressure curve, then, MW and related indices were calculated using a combination of echocardiographic strain data and a noninvasively estimated LV pressure curve [13, 14]: (1) GWI: global myocardial work index, means the area within the global LV pressure-strain loop, calculated from mitral valve closure to mitral valve opening. (2) GCW: global constructive work, means work performed by the LV contributing to LV ejection during systole, estimated the work by the LV segments shortening during systole plus lengthening during isovolumic relaxation. (3) GWW: global wasted work, means work performed by the LV that does not contribute to LV ejection, estimated the work by the LV segments lengthening during systole plus shortening during the isovolumic relaxation phase. (4) GWE: myocardial work efficiency, GWE = GCW/(GCW + GWW) (Fig. 1 C–F).

### Statistical analysis

All data analyses were performed using SPSS 25.0 software (SPSS, Chicago, IL, USA). The Shapiro–Wilk test or Kolmogorov–Smirnov test was used to detect the normality of all values. Differences between the T2DM patients and normal controls were compared with an independent Student′s t-test because the data distribution was normal. For variables with a nonnormal distribution, the nonparametric Mann–Whitney test was used. We defined GLS and global MW values in the normal controls as the normal state and considered the values of T2DM patients to be abnormal. These values in T2DM patients were determined from receiver operating characteristic (ROC) curve analysis by MedCalc software. Youden’s index was selected as the cut-off point that can give the best composite of specificity and sensitivity. Correlations among LVEF, fasting plasma glucose, HbA1c and GLS and global MW values were tested using Pearson or Spearman correlation tests as appropriate. The categorical variables are presented as frequencies and percentages. Data are presented as the mean ± standard deviation (SD) if normally distributed, and as medians and interquartile range boundaries otherwise. A p-value < 0.05 was considered significant in all tests.

### Reproducibility and repeatability

Intraobserver and interobserver variability for GLS and global MW values were determined by repeating measurements in 25 patients randomly selected from among all enrolled patients.

## Results

### Patient characteristics

A total of 120 patients met the baseline inclusion criteria. Nineteen patients were excluded from the strain and MW analyses because of inadequate image quality (n = 12), tachycardia (n = 7) or irregular heartbeat (n = 3). A total of 98 patients were therefore evaluated in the study and were initially divided into two groups: normal controls (n = 50, mean age, 50.38 ± 15.27 years, 25 men) and T2DM patients (n = 48, mean age, 53.79 ± 11.24 years, 25 men).

### Comparison of the patient characteristics and conventional echocardiographic parameters of normal controls and T2DM patients (Table 1)

**Table 1:** Baseline clinical characteristics, conventional two-dimensional echocardiographic parameters between T2DM patients and normal subjects

Variable	Normal (50)	T2DM (48)	p-value
Clinical			
Age (yrs)	50.38 ± 15.27	53.79 ± 11.24	0.212
Male (%)	25(50)	25(52)	0.846
BMI (kg/m^2^)	22.92 ± 1.17	22.68 ± 1.44	0.358
Heart Rate (bpm)	73.72 ± 10.74	76.25 ± 9.50	0.221
SBP (mmHg)	121.94 ± 11.60	127.15 ± 12.91	0.038
DBP (mmHg)	76.78 ± 8.33	77.79 ± 8.99	0.565
Fasting plasma glucose (mmol/L)	4.82 ± 0.63	13.28 ± 4.36	< 0.001
HbA1c (%)	5.02 ± 0.62	10.13 ± 2.15	< 0.001
TCH (mmol/L)	3.91 ± 0.64	3.99 ± 0.77	0.585
TG (mmol/L)	1.10 ± 0.30	1.19 ± 0.60	0.365
HDL (mmol/L)	1.27 ± 0.29	1.19 ± 0.36	0.272
LDL (mmol/L)	2.06 ± 0.50	2.27 ± 0.62	0.060
Medical treatment in hospitalization (%)			
Diet treatment	0 (0)	0 (0)	
Oral drug	0 (0)	12 (25)	
Insulin	0 (0)	26 (54)	
Insulin + Oral drug	0 (0)	10 (21)	
Echocardiography			
LAd (mm)	33.50 ± 3.09	35.46 ± 3.91	0.007
IVSd (mm)	8.85 ± 0.87	9.35 ± 1.23	0.021
LVPWd (mm)	8.54 ± 0.95	9.04 ± 1.15	0.021
LVDd (mm)	46.30 ± 2.52	46.96 ± 3.68	0.302
LVEDV (ml)	74.54 ± 13.47	72.52 ± 14.07	0.470
LVESV (ml)	25.80 ± 6.32	27.44 ± 5.61	0.179
LVEF (%)	65.59 ± 4.01	62.06 ± 4.87	< 0.001
E (m/s)	0.88 ± 0.13	0.79 ± 0.13	< 0.001
A (m/s)	0.68 ± 0.14	0.69 ± 0.19	0.924
E/A	1.34 ± 0.30	1.22 ± 0.34	0.079
e′ (m/s)	0.13 ± 0.03	0.09 ± 0.02	< 0.001
a′ (m/s)	0.09 ± 0.02	0.10 ± 0.02	< 0.001
E/e′	7.26 ± 1.60	10.35 ± 2.55	< 0.001

*BMI* body mass index, *SBP* systolic blood pressure, *DBP* diastolic blood pressure, *TCH* total cholesterol, *TG* triglyceride, *HDL* high density lipoprotein, *LDL* low density lipoprotein, *LAD* left atrial diameter, *IVSD* interventricular septal thickness in end-diastolic period, *LVPWD* left ventricular posterior wall thickness in end-diastolic period, *LVDD* left ventricular diameter in end-diastolic period, *LVEDV* left ventricular end-diastolic volume, *LVESV* left ventricular end-systolic volume, *LVEF* left ventricular ejection fraction, *E* peak velocity during early diastole of anterior mitral valve, *A* peak velocity during late diastole of anterior mitral valve, *e′* peak early diastolic annular velocities using TDI, *a′* peak late diastolic annular velocities using TDI

The values of SBP, fasting plasma glucose and HbA1c were significantly higher in the T2DM patients than in normal controls (p < 0.05). There were no significant differences in age, BMI, HR, sex, DBP, TCH, TG, HDL and LDL between the normal controls and T2DM patients (P > 0.05).

The values of LAd, IVSd, LVPWd, a′ and E/e′ in the T2DM patients were significantly higher than those in normal controls (p < 0.05), however, the LVEF, E and e′ values in the T2DM patients were significantly lower than those in normal controls (p < 0.05). There were no significant differences in levels of LVDd, LVEDV, LVESV, A or E/A between the normal controls and T2DM patients (p > 0.05).

### LV GLS and global MW in normal controls and T2DM patients (Table 2 and Fig. 2)

**Table 2:** GLS and Myocardial work between T2DM patients and normal subjects

Variable	Normal (50)	T2DM (48)	p-value
GLS (%)	− 22.54 ± 1.72	− 19.66 ± 2.25	< 0.001
MW			
GWI (mmHg%)	2282.82 ± 286.11	2046.40 ± 321.22	< 0.001
GCW (mmHg%)	2659.68 ± 322.20	2417.60 ± 359.56	0.001
GWW (mmHg%)	64.00 (46.00–90.50)	86.00 (61.25–121.75)	0.002
GWE (%)	97.00 (96.00–98.00)	96.00 (94.25–97.00)	0.001

*GLS* global longitudinal strain, *MW* myocardial work, *GWI* global myocardial work index, *GCW* global constructive myocardial work, *GWW* global wasted myocardial work, *GWE* global myocardial work efficiency

**Fig. 2:**
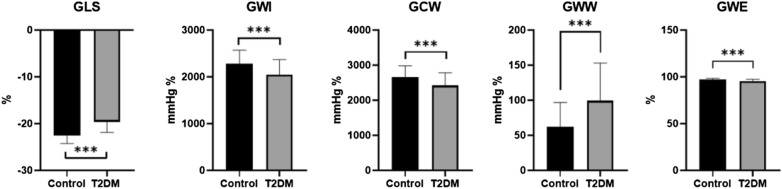
GLS, GWI, GCW, GWW and GWE between normal controls and T2DM patients (***means p < 0.001)

The values of LV GLS in T2DM patients were significantly lower than those in normal controls (p < 0.001). The GWW value was significantly higher in T2DM patients than in normal controls, however, the GWI, GCW and GWE value was significantly lower in T2DM patients than in normal controls (p < 0.001).

### ROC analysis (Table 3 and Fig. 3)

**Table 3:** Receiver operating characteristic curve analysis for the detection LV dysfunction of T2DM patients

Variable	GLS (%)	GWI (mmHg%)	GCW (mmHg%)	GWW (mmHg%)	GWE (%)
AUC (SE)	0.840	0.689*	0.683*	0.734	0.784
AUC (95%CI)	0.753–0.907	0.588–0.779	0.581–0.773	0.636–0.819	0.689–0.861
Cut-off value	− 21.1	2024	2467	46	97
Sensitivity	75	50	58	96	94
Specificity	80	82	74	46	54
Youden index	0.5500	0.3200	0.3233	0.4183	0.4775

^*^Significantly different (p < 0.05) when global MW values compared with the GLS

**Fig. 3:**
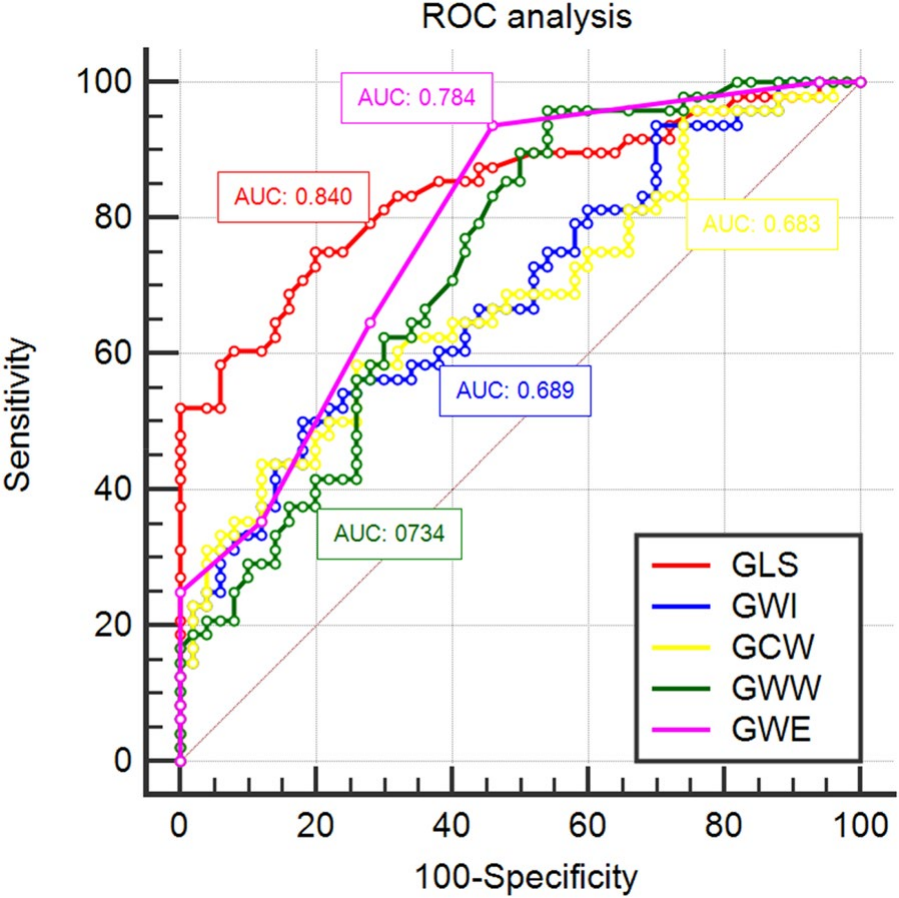
ROC analysis for detecting the accuracy of subclinical LV dysfunction in T2DM patients

ROC curve analysis was used to discriminate whether global MW parameters and GLS were able to predict subclinical LV myocardial dysfunction in T2DM patients. According to the ROC analysis, the area under the curve (AUC) of GLS was 0.840, the cut-off value was − 21.1%, with a sensitivity of 75% and specificity of 80%. The AUC of GWI was 0.689, the cut-off value was 2024 mmHg%, with a sensitivity of 50% and specificity of 82%. The AUC of GCW was 0.683, the cut-off value was 2467 mmHg%, with a sensitivity of 58% and specificity of 74%. The AUC of GWW was 0.734, the cut-off value was 46 mmHg%, with a sensitivity of 96% and specificity of 46%, and the AUC of GWE was 0.784, the cut-off value was 97%, with a sensitivity of 94% and specificity of 54%. The comparation of AUCs showed that there was no significant difference between GWW, GWE and GLS (p > 0.05).

### Correlation tests among LVEF, fasting plasma glucose, HbA1c, GLS and MW in T2DM patients (Table 4 and Figs. 4 and 5)

**Table 4:** Correlation tests among fasting plasma glucose, LVEF, HbA1c, GLS and global MW in T2DM patients

Variable	GWI		GCW		GWW		GWE	
	r	*p*-value	r	*p*-value	r	*p*-value	r	*p*-value
Fasting plasma glucose	− 0.005	0.971	0.059	0.689	0.410	0.004	− 0.392	0.006
HbA1c	− 0.122	0.409	− 0.091	0.539	0.224	0.125	− 0.174	0.238
LVEF	0.235	0.109	0.150	0.310	− 0.141	0.340	0.204	0.165
GLS	− 0.343	0.017	− 0.516	< 0.001	− 0.059	0.692	− 0.138	0.351

**Fig. 4:**
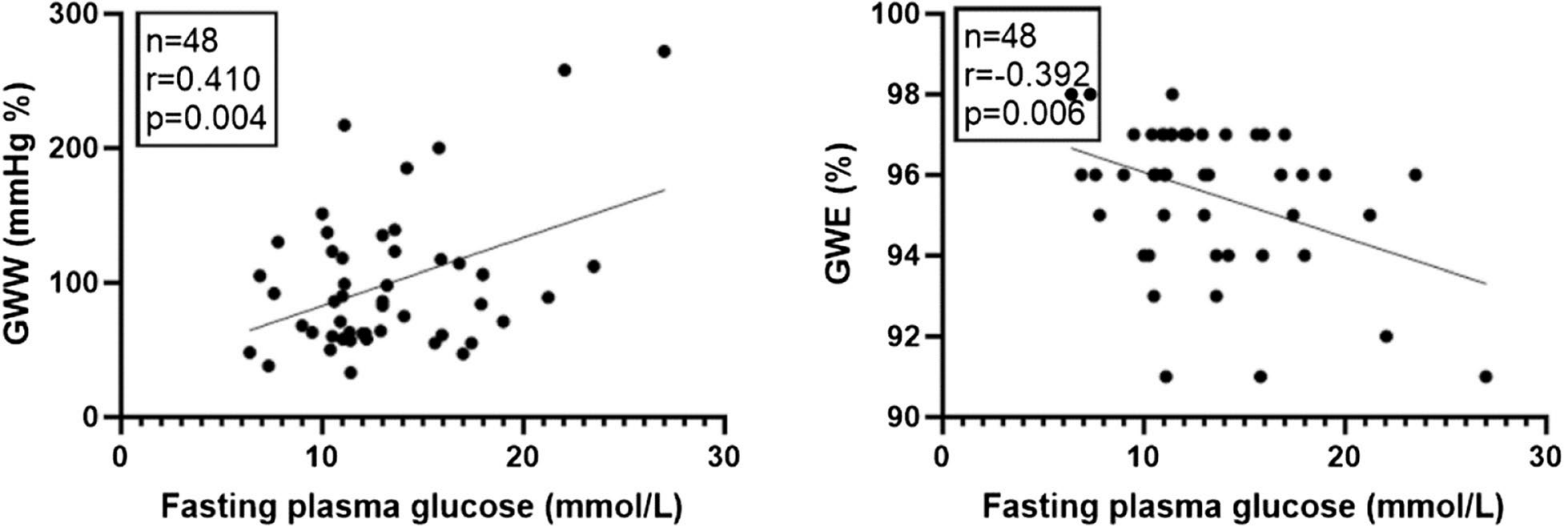
Correlation tests showed in T2DM patients, fasting plasma glucose was positively correlate with GWW, while negatively correlate with GWE

**Fig. 5:**
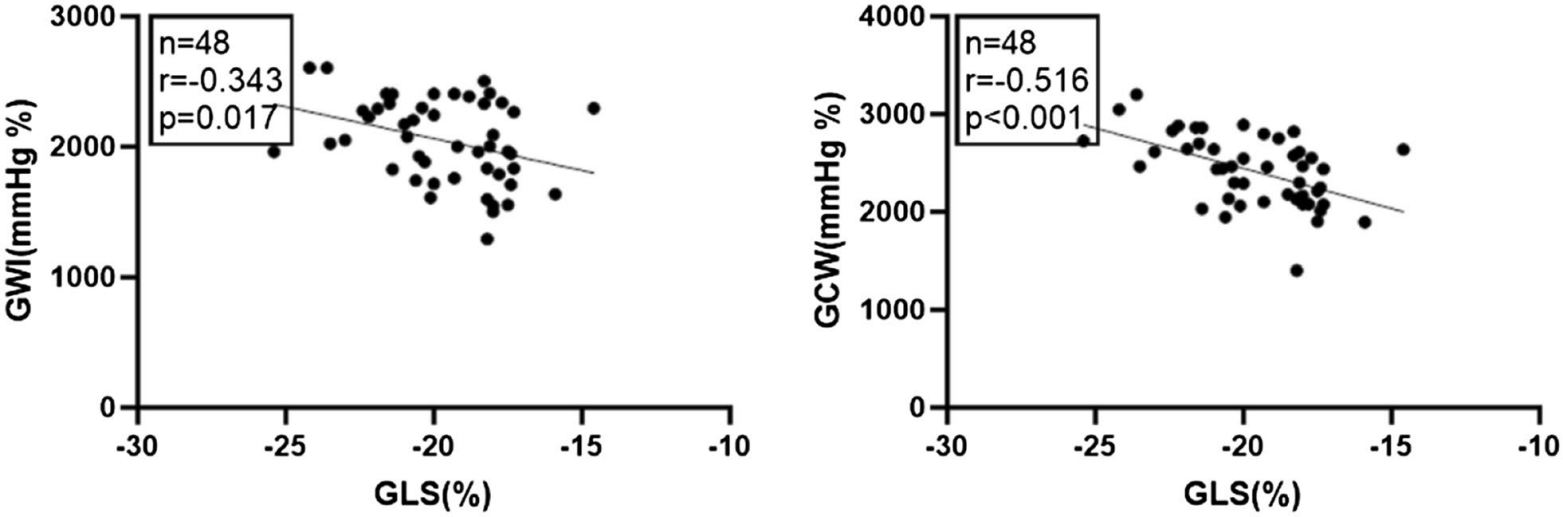
Correlation tests showed in T2DM patients, GLS was negatively correlated with GWI and GCW

In T2DM patients, fasting plasma glucose was positively correlated with GWW but negatively correlated with GWE. GLS was negatively correlated with GWI and GCW.

### Intra- and interobserver variability (Table 5)

**Table 5:** ICCs for intra- and interobserver variability for GLS and MW parameters

Variable	Interobserver variability		Intraobserver variability	
	ICC	95% CI	ICC	95% CI
GLS (%)	0.911	0.809–0.960	0.923	0.834–0.965
GWI (mmHg %)	0.926	0.839–0.967	0.932	0.851–0.969
GCW (mmHg %)	0.914	0.815–0.961	0.878	0.743–0.944
GWW (mmHg %)	0.996	0.991–0.998	0.987	0.971–0.994
GWE (%)	0.973	0.939–0.998	0.967	0.926–0.985

Intra- and interobserver variabilities were assessed via the intraclass correlation coefficients (ICCs) for the measurements. GLS and global MW parameters showed excellent intra- and interobserver correlations, with ICC values > 0.85.

## Discussion

The main finding of the study was that subclinical LV myocardial dysfunction was detected by global MW in T2DM patients with preserved LV systolic function.

Cardiac impairment by T2DM includes microvascular impairment, metabolic disturbance, subcellular component abnormalities, cardiac autonomic dysfunction, and a maladaptive immune response. Eventually, functional and structural changes in myocardium without coronary artery disease are caused by T2DM, and this is called diabetic cardiomyopathy [15]. Diabetic cardiomyopathy may first induce diastolic dysfunction and then systolic dysfunction. Finally, clinical heart failure may occur. With the development of imaging examinations, the detection of cardiac function has become increasingly easy. Echocardiography for measuring MW is considered the newest tool for the assessment of LV systolic dysfunction. Billig et al. [16] evaluated left and right ventricular structure, function and myocardial work by transoesophageal echocardiography (TEE) in swine and found that myocardial contractility and mechanics could be reliably evaluated with noninvasive GWI derived from echocardiography without additional invasive measures. Galli et al. [17] provided reference values for GWI, GCW, GWW and GWE in a group of healthy volunteers controlling for age and sex and found that assessment of MW was feasible in normal subjects. The presented referral ranges of GWI, GCW, GWW and GWE were not affected by age. According to previous studies, MW has been reported in many diseases, including coronary heart disease [14], hypertension, cardiac resynchronization therapy (CRT), percutaneous coronary intervention (PCI) [18], aortic stenosis [19], transcatheter aortic valve replacement (TAVR) [20], dilated cardiomyopathy [21], hypertrophic cardiomyopathy [22], chronic kidney disease [23], and cardiac amyloidosis [24]. However, there has been little research about LV systolic function in T2DM patients with MW.

Liu et al. [25] examined the prognostic value of GLS in T2DM patients and found that in T2DM patients without any history of cardiovascular disease, impaired GLS was associated with cardiovascular events. Our research was consistent with this research, and we found that LV GLS was decreased in the T2DM patients compared with normal controls. MW analysis showed that the GWW value was significantly higher in T2DM patients than in normal controls and that the GWI, GCW and GWE value was significantly lower in T2DM patients than in normal controls. The results may indicate that subclinical LV myocardial dysfunction was present in T2DM patients, although LV systolic function was normal. We considered that the results were related to the pathological changes of the LV myocardium in T2DM. In T2DM, persistent hyperglycaemia causes molecular and metabolic changes in cardiomyocytes and damages the coronary microvasculature. Hypoxia of cardiomyocytes and ischaemia results in myocardial hypertrophy, perivascular and fibrosis, LV stiffness, and systolic and diastole dysfunction in T2DM [15]. Normal myocardial fibres consist of longitudinal and circumferential myocardial fibres. Almost 70% of the myocardial fibre is longitudinal, and 30% is circumferential. If cardiomyocyte apoptosis, myocardial fibrosis and necrosis occur in T2DM, the sequence of the myocardium may change, eventually leading to damage to myocardial systolic function.

ROC analysis showed that GWW and GWE had the same diagnostic efficacy with GLS. The results may indicate global MW can evaluate the subclinical LV dysfunction in T2DM patients accurately.

In T2DM patients, fasting plasma glucose was positively correlated with GWW but negatively correlated with GWE, and GLS was negatively correlated with GWI and GCW. However, the correlations were not strong (0.3–0.6) and they could not accurately predict the subclinical LV myocardial dysfunction in T2DM patients.

## Conclusion

From the research, we found that global MW as new technique could detect the subclinical LV myocardial dysfunction and confirm that the impaired LV function in T2DM patients with preserved LV systolic function.

## Limitations

First, the number of enrolled subjects in this study was small and it is also a single center study, in the future research, we will expand the sample size to makes the research more reliable. Second, 2D-STE has the shortcoming of the speckle “out of plane motion”, if the speckles move out of plane during contraction, the software cannot be tracked successfully. Third, patients with COPD were insufficient and excluded for the analysis.

## Data Availability

The datasets used and analyzed during the current study are available from the corresponding author on reasonable request.

## Abbreviations

LV: Left ventricle
LVEF: Left ventricle ejection fraction
T2DM: Type 2 diabetes mellitus
GLS: Global longitudinal strain
MW: Myocardial work
PSL: Pressure-strain loops
GMW: Global myocardial work
GWI: Global myocardial work index
GCW: Global constructive work
GWW: Global wasted work
GWE: Myocardial work efficiency
STE: Speckle-tracking echocardiography
TDI: Tissue Doppler imaging
HR: Heart rate
BMI: Body mass index
SBP: Systolic blood pressure
DBP: Diastolic blood pressure
TCH: Total cholesterol
TG: Triglyceride
HDL: High density lipoprotein
LDL: Low density lipoprotein
LAD: Left atrial diameter
IVSd: Interventricular septal thickness in end-diastolic period
LVPWd: Left ventricular posterior wall thickness in end-diastolic period. LVDd: LV diameter in end-diastole period
LVEDV: Left ventricular end-diastolic volume
LVESV: Left ventricular end-systolic volume
HbA1c: Glycated haemoglobin
ROC: Receiver operating characteristic
AUC: Area under the curve
ICCs: Intraclass correlation coefficients
CRT: Cardiac resynchronization therapy
PCI: Percutaneous coronary intervention
TAVR: Transcatheter aortic valve replacement
